# Intimate partner violence and stress-related disorders: from epigenomics to resilience

**DOI:** 10.3389/fgwh.2025.1536169

**Published:** 2025-05-12

**Authors:** Anna Carannante, Marco Giustini, Federica Rota, Paolo Bailo, Andrea Piccinini, Gabriella Izzo, Valentina Bollati, Simona Gaudi

**Affiliations:** ^1^ Department of Environment and Health, Italian Institute of Health, Rome, Italy; ^2^ EPIGET—Epidemiology, Epigenetics and Toxicology Lab, Department of Clinical Sciences and Community Health, University of Milan, Milan, Italy; ^3^ Section of Legal Medicine, School of Law, University of Camerino, Camerino, Italy; ^4^ Department of Biomedical Sciences for Health, Università Degli Studi di Milano, Milan, Italy; ^5^ Service for Sexual and Domestic Violence (SVSeD), Fondazione IRCCS Ca’ Granda Ospedale Maggiore Policlinico, Milan, Italy; ^6^ Press Office, Italian Institute of Health, Rome, Italy; ^7^ Occupational Health Unit, Fondazione Irccs Ca’ Granda Ospedale Maggiore Policlinico, Milan, Italy

**Keywords:** intimate partner violence, stress-related disorders, epigenetics, vesicles, microbiome, resilience

## Abstract

Intimate Partner Violence (IPV) is a major public health problem to be addressed with innovative and interconnecting strategies for ensuring the psychophysical health of the surviving woman. According to the World Health Organization, 27% of women worldwide have experienced physical and sexual IPV in their lifetime. Most of the studies on gender-based violence focus on short-term effects, while long-term effects are often marginally included even though they represent the most serious and complex consequences. The molecular mechanisms underlying stress-related disorders in IPV victims are multiple and include dysregulation of the hypothalamic-pituitary-adrenal axis, inflammatory response, epigenetic modifications, neurotransmitter imbalances, structural changes in the brain, and oxidative stress. This review aims to explore the long-term health consequences of intimate partner violence (IPV), emphasizing the biological and psychological mechanisms underlying stress-related disorders and resilience. By integrating findings from epigenetics, microbiome research, and artificial intelligence (AI)-based data analysis, we highlight novel strategies for mitigating IPV-related trauma and improving recovery pathways. Genome-wide environment interaction studies, enhanced by AI-assisted data analysis, offer a promising public health approach for identifying factors that contribute to stress-related disorders and those that promote resilience, thus guiding more effective prevention and intervention strategies.

## 1 Introduction

Intimate partner violence (IPV) is a critical public health issue that crosses socioeconomic, cultural, and geographic boundaries. Defined as any behavior perpetrated against a current, or previous, intimate partner that causes physical violence, emotional abuse, sexual violence, controlling and coercive behavior, and any action perpetrated against a current, or previous, intimate partner that causes physical, mental, or sexual harm (1), IPV includes acts of physical aggression, sexual coercion, psychological abuse, and controlling behavior. According to the World Health Organization (WHO), in 2018 between 736 million and 852 million women worldwide aged 15 years and older experienced physical and/or sexual violence by a current or former husband or male intimate partner, or sexual violence by a non-partner (e.g., strangers, acquaintances, friends) at least once in their lifetime (1).

In Italy, 38.1% of violence against women that results in at least one emergency department attendance is perpetrated by a spouse or partner (2). In addition, there are an estimated 1.9 million women between the ages of 15 and 49 in Italy who have experienced one or more episodes of violence by a partner in their lifetime. Of these, about 466,000 have experienced violence in the past 12 months (1).

These estimates confirm that physical and sexual IPV and non-partner sexual violence remain pervasive in the lives of women and young girls worldwide.

The impact of IPV transcends immediate physical injuries, encompassing permanent psychological trauma, chronic health conditions, and significant socioeconomic consequences. Survivors often face a range of negative outcomes, including post-traumatic stress disorder (PTSD), depression, anxiety, dissociation, substance abuse, and weakened economic opportunities. Dissociative symptoms in particular have been identified as significant mediators between domestic violence exposure and suicidal ideation (3–5). In addition, IPV extends a cycle of violence affecting future generations; children who witness IPV are at increased risk of developing behavioral and emotional disorders and may become perpetrators or victims of violence themselves (6, 7).

Despite its widespread incidence, IPV is still underreported and inadequately addressed in many countries (8, 9). Cultural norms, stigma, fear of retaliation, and limited resources often contribute to the silence surrounding IPV, hindering efforts to support survivors and hold perpetrators accountable. The COVID-19 pandemic has exacerbated this condition, with measures of isolation and economic stressors leading to an increase in IPV cases worldwide (10).

Understanding the dynamics of IPV is critical to developing holistic and sustainable solutions that mitigate its impact and promote a culture of nonviolence and respect in intimate relationships. Despite extensive research on the psychological and social dimensions of IPV, there remains a critical gap in understanding the biological mechanisms that underlie both vulnerability to IPV-related trauma and pathways to resilience. The present narrative review aims to address this gap by exploring the multifaceted nature of IPV and bringing together in a “coherent framework” the experimental evidence that identifies epigenetic modifications and microbiome variations as intermediate molecular steps for achieving resilience. By elucidating these biological mechanisms, we hope to inform more targeted and effective interventions that can break the cycle of violence and promote healing at both the individual and societal levels. This paper examines emerging areas such as (1) the epigenetic signature of IPV, which highlights how trauma can alter gene expression; (2) the role of the brain-gut axis as a cross-link to resilience, illustrating the connection between psychological stress and gut health; (3) the potential use of extracellular vesicles (EVs) as markers of IPV resilience, offering insights into cellular responses to trauma; (4) the sleep disturbances as a symptom of IPV, emphasizing the impact of chronic sleep issues on overall health and recovery.

Our literature search was conducted between June and September 2024 using a custom-made search terms list (see Supplementary Material). Studies were selected based on relevance to the key areas explored in this review as epigenetic signatures of IPV, brain-gut axis in relation to resilience, extracellular vesicles as markers of IPV resilience, and sleep disturbances as symptoms of IPV. We prioritized peer-reviewed articles published within the last five years (more than 50% of the references published between 2019 and 2024), with exceptions made for seminal works that established fundamental concepts in the field.

The PubMed database and websites of institutional sources such as WHO, CDC were queried for article search. For this purpose, Artificial Intelligence (AI) tools have been also used to streamline the literature review process, allowing us to efficiently sort through large datasets and identify relevant studies reducing the risk of overlooking key research.

AI-based language editing tools were used to enhance the clarity and fluency of the manuscript, ensuring it meets the high standards of scientific communication without altering the originality of the ideas. In our study, we used ChatGPT-4 as a supporting tool to enhance the efficiency of literature screening, article summarization, and thematic identification related to IPV and stress-related disorders. ChatGPT-4 was particularly useful in streamlining the initial stages of the review, where it assisted in scanning and summarizing abstracts from PubMed and other academic sources, helping to identify studies that aligned with our research objectives. Additionally, we leveraged ChatGPT-4 to expand our search queries by suggesting alternative keywords and related concepts, ensuring a broader and more comprehensive coverage of relevant literature.

Beyond screening, ChatGPT-4 played a role in summarizing key findings, methodologies, and conclusions of selected studies, allowing us to assess their relevance efficiently. The AI model also helped identify recurring themes across studies, which facilitated the categorization of findings into the broader topics discussed in the manuscript, such as epigenetics, the gut-brain axis, and AI applications in resilience research.

All interpretations, conclusions, and original content were developed independently by the authors. The ethical use of AI was ensured by maintaining full human oversight throughout the whole writing process.

## 2 Epidemiology of IPV

It is estimated that about 27% of women aged 15–49 years worldwide have experienced physical and/or sexual IPV in their lifetime, whilst 13% have been subjected to physical and/or sexual IPV at some point within the past 12 months (1). For lifetime IPV, prevalence ranges from as low as 16% in Southern Europe to as high as 51% in Melanesia. Past-year IPV ranges from 4% in Australia and New Zealand to 32% in Central sub-Saharan Africa (Table 1).

**Table 1:** Prevalence of lifetime and past-year physical and sexual IPV in women aged 15–49 years, by regions.

Regions	Lifetime	Past Year
**Europe**	**23%**	**5%**
Western Europe	21%	5%
Southern Europe	16%	4%
Northern Europe	23%	5%
Eastern Europe	20%	7%
**Africa**	**33%**	**19%**
Northern Africa	31%	15%
Western sub-saharian Africa	27%	15%
Southern sub-saharian Africa	27%	14%
Central sub-saharian Africa	44%	32%
Eastern sub-saharian Africa	37%	24%
**Americas**	**25%**	**9%**
Northern America	27%	6%
Central latin America	24%	10%
Caribbean	21%	9%
Andean latin America	38%	12%
Tropical latin America	23%	7%
Southern latin America	26%	6%
**Asia**	**27%**	**13%**
Central Asia	18%	9%
Southern Asia	35%	19%
Eastern Asia	20%	8%
South-Eastern Asia	21%	9%
Western Asia	29%	13%
**Oceania**	**31%**	**10%**
Australia and New Zealand	25%	4%
Melanesia	51%	30%
Micronesia	42%	22%
Polynesia	39%	19%

In bold the weighted average percentage for the continental regions.

Based on data from the European Injury Database (EU-IDB), the epidemiologic surveillance system of injuries and violence, in 38.7% of Emergency Departments attendances for violence against women, the perpetrator was the spouse or partner. Of these cases, 67.0% and 6.7% were physical and sexual assault respectively (2).

According to the National Intimate Partner and Sexual Violence Survey 47.3% of women and 40.0% of male (i.e., 59 million and 52 million respectively) in the United States reported any contact sexual violence, physical violence, and/or stalking victimization by an intimate partner at some point in their lifetime. In the 12 months prior to the survey, 7.3% of women (or 9 million) and 6.8% of men (or 8 million) reported any contact sexual violence, physical violence, and/or stalking by an intimate partner (11).

As reported by a recent systematic review (12), out of 201 studies included with 250,599 women, primarily from high-income countries, the prevalence of having experienced any IPV in the past year was 24.2% (95% CI 20.4%–28.4%). Stratifying by different types of IPV (e.g., emotional psychological violence, physical violence, sexual violence, controlling behavior, and harassment), higher prevalence rates were reported for psychological violence 27.0% (95% CI: 22.1%–32.4%), whilst the lowest prevalence was reported for physical violence (15.7%, 95% CI: 12.8%–19.1%), and sexual IPV (10.1%, 95% CI: 7.6%, 13.2%). Finally, the pooled prevalence of lifetime any IPV was 37.3% (95% CI: 30.6%–44.6%).

A large-sample survey carried out in Spain revealed that 32.4% of the female had experienced IPV. Moreover, in a total of 9,568 interviewed women survivors of IPV, 27% suffered psychological control violence, 23.2% had psychological emotional violence, 11% reported that they had suffered physical violence, 8.9% reported they had suffered sexual violence, (13).

According to Dardis et al. (14), women reported an average of 27.9 (SD ± 60.5) acts of IPV within the past year. Most of these acts were psychological (mean = 18.51, SD ± 28.58), with lower means for physical (mean = 4.97, SD ± 22.19) and sexual IPV (mean = 3.40 SD ± 16.10).

In particular, the higher prevalence of IPV was observed among younger individuals, particularly those aged 18–24. From a recent study by the WHO, published in The Lancet Child & Adolescent Health, emerges that one in six adolescents with a partner has been the victim of physical or sexual violence and one in 4 (around 19 million) will be a victim before the age of 20 (15).

IPV is also present among older adults, though often underreported. Higher rates of IPV were observed among African American and native American women compared to white women. For Hispanic women, similar rates of IPV were observed as non-Hispanic white women but with variations based on acculturation and immigration status (16).

IPV not only causes pain and suffering to the victims, but also places enormous costs on the economy and society. However, the extent and associated costs of IPV, encompassing lost economic output, public spending on health, legal and social problems, specialized services to mitigate harms, and personal impacts on victims, are rarely evaluated. According to a recent report of the European Institute for Gender Equality report (17), the estimated cost per year of IPV against women in the EU-27 was nearly EUR 152 billion, representing 87% of all costs of IPV against both women and men.

Based on 43 million U.S. adults with victimization history, the estimated IPV lifetime cost was $103,767 per female victim and $23,414 per male victim, or a population economic burden of nearly $3.6 trillion (2014 US$) over victims' lifetimes (18).

## 3 Intimate partner violence and stress related disorders

IPV is a public health crisis with devastating effects on individuals, families, and communities and, is the form of violence most experienced by women globally associated with physical, mental, sexual, and reproductive health problems and death (from homicide and suicide). Increasingly, technology is being used to facilitate abuse, including abuse on social media and other online platforms, installation of stalker ware on personal devices, and manipulation of smart meters, locks, and cameras (19–21).

In terms of frequency and types of acts, IPV occurs along a continuum of severity and as a combination of forms of violence (physical, sexual, emotional, and controlling behavior). Some researchers considered intimate terrorism, a mechanism by which violence reinforces control over a partner (22) and situational violence (where violence is a physical response to anger or frustration, but domination is not the motivation), and the first arguably requires more substantial intervention than the latter (23). In terms of frequency, severity, and combination of violence used, another approach considers violence as occurring along a continuum of escalation (24) requiring different intervention points and responses tailored to different patterns of abuse.

The effects can be profound and long-lasting, often requiring professional intervention and support. The relationship between IPV and psychological and psychiatric health conditions such as PTSD, depression, and anxiety were observed. In particular, PTSD is two to three times more common in women than in men (25, 26) and may occur in individuals who have experienced or witnessed traumatic events, characterized by symptoms such as intrusive thoughts, hyperarousal, avoidance behaviors, negative alterations in mood and cognition, flashbacks, nightmares, severe anxiety, uncontrollable thoughts about the event, with high prevalence, among IPV victims, due to the chronic and traumatic nature of abuse (27).

Numerous studies have documented high rates of PTSD among IPV survivors. Research indicates that between 31% and 84% of women who experience IPV develop PTSD, a prevalence significantly higher than that observed in the general population (28). More recent studies indicate a prevalence of PTSD in women victims of IPV ranging from 31% and 58% percent (29–31).

Moreover, IPV victims are at a higher risk of developing depression, often due to constant stress and emotional abuse, characterized by persistent sadness, loss of interest in activities, changes in appetite and sleep, feelings of worthlessness. In addition, the fear and control exerted by the abuser can lead to various anxiety disorders consisting in excessive worry, panic attacks, phobias, social anxiety (27). Then, to consider that IPV significantly raises the risk of suicidal ideation and behaviors due to the overwhelming emotional distress, the dependence on alcohol or drugs to cope with emotional pain and trauma, negative self-perception, feelings of inadequacy, physical symptoms without a medical cause, such as chronic pain, gastrointestinal issues. Finally, control problems and stress from IPV can contribute to the development of eating disorders, as anorexia, bulimia, binge eating (32).

A comprehensive approach encompassing mental health and molecular mechanism, can significantly improve the physical and psychological well-being of those affected by IPV.

Understanding the molecular processes on IPV is critical to developing effective intervention and support systems for victims.

The molecular mechanisms underlying stress-related disorders in IPV victims are multiple and include dysregulation of the HPA axis, inflammatory response, epigenetic changes, neurotransmitter imbalances, structural changes in the brain, and oxidative stress. The hypothalamic-pituitary-adrenal (HPA) axis has been known as the primary mediator of stress (33, 34). However, stressors also elicit responses from the limbic and frontal cortex and other regions of the nervous system and associated organs. The HPA axis mediates the release of hormones such as corticotropin-releasing hormone (CRH), which is involved in several physiological processes (35).

Furthermore, the current concept of stress involves allostatic mechanisms of dysregulation and inflammatory responses to non-negligible noxious stimuli that underlie the pathophysiology of stress-related disorders (36).

Subsequently, both animal and human data demonstrated that stress-induced inflammatory reactions are the underlying pathophysiological process in stress-related diseases. The level of inflammatory biomarkers, tumor necrosis factor (TNF)-α, interleukin (IL)-6 and C-reactive protein (CRP) among people with anxiety disorders have been studied by observing how they interact with neurotransmitter systems, especially serotonin and dopamine, it influences mood and behavior (37).

In fact, chronic stress, associated with IPV, can alter the dopaminergic system, affecting motivation and emotional regulation, and this can manifest in symptoms of depression and PTSD (38). Meanwhile, victims of IPV have disturbances in the serotonin system, which is crucial for mood regulation. Reduced serotonin availability or receptor sensitivity is linked to depression and anxiety (39). The balance between excitatory neurotransmission (glutamate) and inhibitory neurotransmission (gamma-aminobutyric acid -GABA), is also often affected by chronic stress and leads to symptoms commonly observed in PTSD, (40).

In the last years, new experimental data suggest that stress plays an important role in the disruption of the blood-brain barrier (BBB), a neurovascular unit that controls the movement of substances into the brain parenchyma and immune cells in the blood and prevents disturbances to brain substances from the periphery (33, 41–43).

Identify biomarkers that distinguish between persons at high and low risk of developing PTSD after trauma exposure could be address the better interventions to the high-risk groups that are at risk in the long term to rising the non-communicable diseases associated to violence.

## 4 Epigenetic signature of IPV

The correlation between violence and epigenetics is an emerging multidisciplinary field of research that explores how environmental factors such as stress, abuse, and violence can influence gene expression through epigenetic modifications. Pioneering studies have demonstrated associations between IPV and changes in DNA telomere length (44), as well as inflammatory and immune responsiveness, including alterations in CRP levels and proinflammatory (IL-6, TNF-α) and anti-inflammatory (IL-10) cytokines. IPV has also been linked to dysregulation of the HPA axis (45).

The HPA axis is a crucial system that helps the body respond to stress. During typical short-term stress, the HPA axis activates the “fight-or-flight” response, releasing hormones like cortisol to manage stress (46). Cortisol helps reduce non-essential functions, allowing the body to focus on the immediate threat. This process is regulated by a negative feedback loop, where cortisol signals the body to dial back the stress response once the threat has passed (47).

Chronic stress, however, leads to an increased allostatic load, the “wear and tear” on the body caused by prolonged stress (46). This extended exposure can cause the HPA axis to malfunction, resulting in irregular cortisol levels (48). Research indicates that while the body initially increases cortisol production during early trauma or stress, prolonged exposure eventually leads to a drop-in cortisol level (49). This imbalance in cortisol can contribute to various health issues, including metabolic disorders and cardiovascular risks (50).

Studies have shown that early trauma can lead to long-lasting epigenetic changes, with evidence from animal models indicating that the offspring of stressed rodents can exhibit similar epigenetic alterations in genes associated with the stress response (51). For instance, methylation of the glucocorticoid receptor gene (NR3C1) has been linked to experiences of childhood abuse, affecting the stress response in adulthood (52) Provenzi et al. (53) examined epigenetic changes in children exposed to abuse and neglect, finding alterations in DNA methylation that may predispose them to emotional and behavioral disorders.

Epigenetic changes have also been observed in genes associated with aggression and stress response in individuals exposed to violence. Increased methylation in the monoamine oxidase A (MAOA) gene promoter, for instance, has been linked to aggressive behaviors in those who experienced childhood abuse (54). Furthermore, evidence suggests that traumatic experiences can be epigenetically transmitted across generations (51). De Bellis et al. (55) found alterations in DNA methylation in genes associated with stress response and emotional regulation in patients with PTSD, a common consequence of violence. Ferrari et al. (56) explored the impact of domestic violence on Italian women's mental health, indicating that chronic stress can lead to epigenetic changes influencing depression and anxiety risk.

In 2016, ISS collaborated with the University of Milan and the Cà Granda Foundation of the Ospedale Maggiore Policlinico di Milano on a pilot study, “Epigenetics for Women (EpiWE),” aimed at identifying epigenetic markers associated with PTSD resulting from relationship and sexual violence. The EpiWE study was a preliminary attempt to link PTSD and stress-related disorders in women exposed to IPV and sexual violence to epigenetic changes detected in DNA samples. The study identified differential hypermethylation in three genes—brain-derived neurotrophic factor (BDNF), dopamine receptor D2 (DRD2), and insulin-like growth factor 2 (IGF2)—suggesting that violence may interfere with genomic plasticity and gene expression regulation. These findings are promising for identifying epigenetic markers in genes mediating brain plasticity and modulating learning and memory in response to IPV and violence-induced PTSD. Understanding the epigenetic signatures underlying PTSD related to violence against women could lead to better treatments and innovative protocols for precision medicine to reduce long-term effects (57).

Research on the correlation between violence and specific epigenetic markers is still developing but provides crucial insights into how traumatic experiences can have lasting effects on mental and behavioral health through epigenetic changes. This field holds the potential to inform new strategies for preventing and treating the psychological and behavioral consequences of violence, enabling a more precise medical approach.

## 5 Brain gut axes as cross-link to resilience

The connection between the brain-gut axis and resilience is an emerging and fascinating field of scientific research. The brain-gut axis refers to the bidirectional communication between the central nervous system (including the brain) and the enteric nervous system (gut), mediated by a series of biological signals involving the nervous, immune, and endocrine systems.

The gut microbiome, a complex and dynamic community of microorganisms residing in the human gastrointestinal tract, is influenced by various factors that can significantly alter its composition, diversity, and function, thereby affecting overall health. Diet, antibiotics and drugs, rural or urban living environments, physical activity, stress levels, and sleep patterns are key factors that shape the gut microbiome (58–61).

Increased microbial diversity is generally associated with improved intestinal and systemic health. Prolonged physical stress, however, can lead to reduced diversity of the intestinal microbiome, promoting the growth of pathogenic or opportunistic bacteria, such as Escherichia and Staphylococcus species, at the expense of beneficial bacteria like Lactobacillus and Bifidobacterium (62–64).

These alterations in the gut microbiome can have several health implications, including increased intestinal permeability, or “leaky gut,” which allows bacteria and toxins to enter the bloodstream, causing systemic inflammation and further reducing microbial diversity. These effects may contribute to gastrointestinal disorders such as irritable bowel syndrome (IBS) and inflammatory bowel disease (IBD), modulate the immune response, increase the risk of chronic infections, and lead to fatigue due to systemic inflammation and gastrointestinal problems.

IPV, encompassing both physical and psychological trauma, can significantly impact the gut microbiome (65). This effect manifests through various mechanisms, including chronic stress, inflammatory responses, and behavioral changes associated with traumatic experiences. Violence can affect the gut microbiome through stress responses, systemic inflammation, and physiological changes.

Chronic trauma, as experienced in IPV, activates the hypothalamic-pituitary-adrenal (HPA) axis, leading to prolonged release of stress hormones such as cortisol (66). This can alter the gut microbiome's composition, reduce microbial diversity, and promote the proliferation of pathogenic bacteria. Trauma and chronic stress can also cause systemic inflammation, further negatively affecting the intestinal microbiome and contributing to the “leaky gut” condition.

Cryan et al. (67) provide a comprehensive review of the microbiota's influence on brain function and behavior, highlighting the pathophysiological consequences of an aberrant gut-brain network. These include severe inflammatory bowel disorders, altered responses to acute and chronic stress and trauma, and altered behavioral states. The study also emphasizes the need for further research to fully explore microbiota-based therapeutic strategies for brain disorders.

Additionally, people exposed to violence may develop eating disorders or altered eating habits, often characterized by nutrient-poor, processed foods, which negatively affect the gut microbiome by reducing beneficial bacteria. Exposure to violence is also frequently linked to increased alcohol and drug consumption, which can drastically alter the gut microbiome and increase intestinal permeability. Poor living conditions associated with violence can further expose individuals to pathogens and reduced hygiene, adversely affecting the microbiome's composition.

Studies on war veterans exposed to violence and PTSD have shown significant alterations in their intestinal microbiome, linking these changes to gastrointestinal symptoms and mental health issues. Similarly, children exposed to violence or abuse exhibit altered gut microbial compositions compared to their unexposed peers, which are often associated with an increased risk of developing mental and physical disorders in adulthood (68).

Research on adults who have experienced domestic violence has also shown changes in the gut microbiome, including reduced microbial diversity and increased inflammation markers (69), leading to health implications such as gastrointestinal disorders (IBS and IBD) (70), anxiety, depression, and PTSD. The microbiome's influence on mood and behavior also contributes to chronic conditions like diabetes, obesity, and cardiovascular disease. Supporting this evidence are pilot studies by Madison and Kiecolt-Glaser (71) and Hemmings et al. (72). The former explores how stress and mood affect the human gut microbiota, highlighting the connection between psychological stress and gut health, while the latter investigates the association between childhood trauma, including domestic violence, and the gut microbiome's composition, suggesting that early traumatic experiences can have lasting effects on the microbiome.

## 6 Extracellular vesicles as markers of IPV resilience

Extracellular vesicles (EVs), including exosomes, microvesicles, and apoptotic bodies, are membrane-bound particles carrying proteins, lipids, RNA, and other molecules, and playing a crucial role in cell-to-cell communication (73, 74). Research on EVs has grown due to their significant role in various physiological and pathological states. By influencing the behavior of recipient cells, EVs have become important biomarkers for studying health consequences related to IPV.

Recent studies (75–78) suggest that the production and composition of EVs can be affected by stress and trauma, such as those experienced by IPV victims. These alterations may reflect the body's response to ongoing stress, inflammation, and psychological trauma, offering insights into the biological impact of IPV at the cellular level (79). EVs may carry stress-related molecules, such as cortisol or inflammatory cytokines, illustrating how the body responds to chronic stress (80, 81). Furthermore, EVs can transport microRNAs (miRNAs) that regulate gene expression (82, 83). Changes in miRNA profiles within EVs have been linked to the epigenetic modifications observed in IPV survivors, such as alterations in DNA methylation or histone modifications, potentially contributing to conditions like PTSD or depression (84–86).

EVs also play a critical role in the inflammatory response by transporting cytokines and other inflammatory mediators (87). Elevated levels of these mediators in EVs can contribute to systemic inflammation, commonly associated with IPV, and can lead to numerous health issues, including cardiovascular disease and metabolic disorders (88).

The unique properties of EVs also suggest their potential for diagnostic and therapeutic applications in IPV research and treatment (89, 90). Analyzing EV content from body fluids such as blood or saliva can help identify biomarkers associated with IPV, leading to the development of diagnostic tests that detect the physiological and psychological effects of violence (91). Additionally, understanding how EVs are altered by IPV may reveal new therapeutic targets (92).

Currently, awareness of the role of EVs in PTSD and resilience, especially in the context of IPV, remains limited. Most existing studies are conducted among war veterans with PTSD (84) and do not specifically address PTSD resulting from IPV. To fill this gap, longitudinal studies should be performed to clarify the role of EVs as biomarkers of PTSD symptoms and to identify biomarkers that can predict those most at risk of developing severe PTSD symptoms over time.

Key challenges in this field include the standardization of isolation and characterization methodologies, understanding the dynamics of EVs in circulation and brain tissue, and conducting studies with large sample sizes to validate preliminary findings. This new line of research may pave the way toward innovative treatments for PTSD, facilitating clinical interventions before the onset of symptoms and underlying disease processes.

## 7 Sleep disturbances as a symptom of IPV

Triggering hormone responses that make it difficult to fall or stay asleep, stressful life events can raise sleep disturbances by provoking persistent anxiety and/or lasting trauma (93, 94) and generating dysregulation across biological systems through increased allostatic load (95).

According to Hauri and Fisher theoretical framework (96) a traumatic event, including actual and/or perceived threat related to violence, may cause insomnia that subsequently leads to associations of the sleep environment with frustration and arousal, which finally becomes a maintaining factor of the insomnia after the termination of the stressful event (97). This vicious circle is perhaps even more relevant for IPV associated to other stressors and life events, as safety issues may be more acutely felt when one's home and sleep environment (i.e., the bed) represent past or present danger and when one's closest friends and family are (or were) the source of threat (98).

It is well known that sleep disturbances may be a consequence of complex relationships among the environmental, psychological, and physical mechanisms in play for those who suffer IPV. The connection between sleep disruption and IPV has been the focus of some important researchers, finding that IPV victims commonly experience significant sleep disturbances that include truncated sleep, nightmares and less restful sleep (99–103).

Several analytical epidemiological studies have assessed associations of sleep disturbances with IPV victimization after adjusting for multiple confounding factors. The likelihood for stress-related sleep disturbance associated with each type of IPV were 1.24 for physical abuse, 3.44 for sexual abuse, and 2.51 for physical and sexual abuse. The corresponding likelihood for poor sleep quality were: 1.72, 2.82, and 2.50, respectively (104).

These results are in general agreement with other studies that have assessed associations between sleep disturbances and sleep patterns with IPV victimization. Namely, in a study carried out in the general community, was reported that women with history of IPV were associated with a 3.91-fold (95% CI: 1.75–8.73) increased odds of poor sleep quality after controlling for stressful life events, vasomotor symptoms, marital status, annual household income and several other covariates (105).

According to Sanchez et al. (106), after adjustment for maternal age, race, parity, and difficulty paying for basics, women with a history of experiencing IPV in their lifetime had 1.54-fold increased odds of stress-related sleep disturbances (OR 1.54; 95% CI: 1.08–2.17). Stratifying by type of abuse experienced, those who experienced IPV lifetime had elevated odds of stress-related sleep disturbances, both for sexual abuse only (OR = 3.44; 95% CI: 1.07–11.05) and for lifetime physical and sexual abuse (OR = 2.51; 95% CI: 1.27–4.96). Compared with women who had no IPV in the 12 months prior to pregnancy, those who suffered from any IPV in antepartum period had a 2.07-fold increased odds of stress-related sleep disturbances (95% CI: 1.17–3.67). The same study assessed the independent and joint associations of lifetime maternal IPV exposure and antepartum depression with the odds of stress-related sleep disturbance. Compared with women who had no lifetime history of IPV and no antepartum depression (the reference group), women with only antepartum depression (classified as no lifetime history of IPV) had a 3.82 times greater odds of stress-related sleep disturbance (95% CI: 2.27–6.11).

Interestingly, women who had both a history of IPV and antepartum depression were 9.28 times more likely to have stress-related sleep disturbance than the reference group (95% CI: 4.53–19.02). Because the excess odds of stress-related sleep disturbance associated with IPV and antepartum depression was greater than the sum of the excess odds for each risk factor considered independently (2.07 and 3.82 respectively), the authors suggest that there is a more than additive association of IPV and depression on the odds of stress-related sleep disturbance (106).

Lalley-Chareczko et al. (98) analyzed data from a sample of about 35 thousand participants involved in the 2006 Behavioral Risk Factor Surveillance System. According to these results, IPV was assessed in participants for any history of being threatened by, physically hurt by, or forced to have sex with an intimate partner, and, further, as being forced to have sex with or physically injured by an intimate partner within the past year. After adjusting for the effects of age, sex, race/ethnicity, income, education, employment, marital status, physical health and mental health, psychological violence was associated with sleep disturbance (OR = 2.80, *p* < .0001), as was the physical one (OR = 2.68, *p* < .0001), or sexual abuse (OR =  3.24, *p* < .0001). These associations become stronger if the physical or sexual abuse occurred within the past 12 months (OR = 7.74, *p* < .0001 and OR = 7.50, *p* < .0001, respectively).

Several hypotheses have been advanced to explain the observed associations of poor sleep quality with a history of stress and IPV victimization. Stress-related effects on biological rhythms and sleep have been increasingly the focus of related research, suggesting that stress-related sleep and circadian dysregulation may be strongly implicated in the pathophysiology of stress-related disorders and particularly trauma.

The association between traumatic stress and circadian/sleep dysregulation becomes more apparent in PTSD (107). Sleep disruption (e.g., insomnia, nightmares, delayed sleep latency, etc.) represent characteristic clinical symptoms of the disorder with very high prevalence: between 63% and 70% of those who have been diagnosed with PTSD describe insomnia as a significant problem (108).

In IPV survivors, several stress-related biological processes are activated that also impact sleep regulation. Hyperactivation of the SNS due to chronic IPV exposure leads to heightened arousal and inability to relax (109). This hyperarousal impacts the sleep-wake cycle by increasing heart rate, blood pressure, and other physiological markers of stress, making it difficult to fall asleep and stay asleep. As previously mentioned, IPV-related stress and trauma are associated with dysregulation of both serotonin (5-HT) and GABA, which are important in regulating sleep. Decreased serotonin levels are associated with depression and insomnia (110), while GABA deficiency is associated with increased arousal and anxiety, further impacting sleep (111). Ad well as, IPV causes chronic activation of the HPA axis, and prolonged stress leads to elevated cortisol levels, especially at night (112). Excessive cortisol levels disrupt normal sleep patterns and make it difficult to achieve deep, restful sleep. Additionally, elevated levels, in IPV cases, of the inflammatory cytokines (TNF-α), IL-6, and CRP, mentioned above, are associated with both sleep disorders and depression, suggesting an interrelationship between inflammation and sleep. Finally, chronic stress and altered circadian rhythms may lead to decreased melatonin production. Melatonin is a hormone important in regulating the sleep-wake cycle, and reduced melatonin levels in IPV victims may exacerbate insomnia and sleep disorders (113).

Research have consistently shown that insufficient sleep and nightmares occurring soon after trauma exposure predict the onset and persistence of PTSD and other stress-related disorders, including other anxiety disorders, major depression, and addictive disorders (114–116). Similarly, preexisting conditions of insufficient sleep increase the risk of PTSD and other stress-related psychiatric disorders after trauma exposure.

## 8 Artificial intelligence and resilience early signs

A general target of advanced medicine is to increase the effectiveness of treatments while reducing adverse effects. In recent years, this has manifested itself through therapies targeting specific genetic mutations and other “omics” methods. Precision medicine is an emerging approach for disease treatment and prevention that considers individual variability in genes, environment, and lifestyle for each person. Disease-specific biomarkers can steer precision medicine, such as in the context of clinical trials, by selecting study populations that are most likely to benefit from treatment. The use of population-level precision approaches can also improve the targeting of public health interventions.

Precision medicine, through AI techniques, analyzes so-called big data to improve diagnostic capabilities and predictivity of response to therapy to “tailor” treatment to individual characteristics.

Due to the large volume of data available from genomic research, AI techniques have been widely adopted to process this wealth of data and analyze them systematically, thereby revealing patterns, and predict outcomes (117). AI has revolutionized all health disciplines, gaining momentum in oncology, cardiology, radiology, and molecular sciences are no exception. AI gives biotechnologists and molecular scientists more powerful and efficient tools to speed up their work. Genetics is an essential aspect of biotechnology and is one of the disciplines that benefits most from AI. Thanks to machine learning algorithms, large collections of genomic and proteomic data can be analyzed to identify genes and enzymes relevant to the synthesis of specific products.

Indeed, genomic and epigenetic data are often characterized by the presence of many parameters (e.g., the human genome has approximately 3.2 billion DNA base pairs containing around 20,000 protein-coding genes.), which often far exceeds the number of samples to be analyzed and are difficult to analyze using traditional techniques. In statistics, this phenomenon is known as “the Curse of Dimensionality” and refers to the various challenges and complications that arise when analyzing and organizing data in high-dimensional spaces. This can lead to a range of issues including multicollinearity, overfitting and computational complexity.

Breaking the curse of dimensionality with AI-based algorithms that can perform integrated analyses of large-scale multi-omics data must be critical to finding useful information for the diagnosis and therapy (118, 119).

Some AI tools and technologies used in omics analysis include: deep neural networks (models for identifying complex patterns in genomic and proteomic data), clustering algorithms (used to group genes or proteins with similar expressions under different conditions), random forests (machine learning algorithms used for the classification and identification of relevant genetic or protein characteristics), and Support Vector Machines-SVM (algorithm used to predict clinical outcomes based on omics data).

One of the most ambitious goals of AI in omics sciences is to integrate genomic, transcriptomic, proteomic and clinical data to develop a holonomic approach to understanding diseases and treatments. The integration of these data through AI can provide a complete picture of an individual's health, enabling the tailoring of therapies to individual molecular profiles.

This personalized approach is not limited to general disease treatment but also holds transformative potential in addressing specific health challenges, such as those stemming from IPV. By combining diverse biological, psychological, and social data, AI can offer unique insights into the long-term health impacts of trauma and stress, further emphasizing its capacity to deliver highly individualized care.

According to the WHO, artificial intelligence (AI) can already be used to improve the speed and accuracy of diagnosis and screening for diseases, assist with clinical care, strengthen health research and drug development, and support diverse public health interventions, including outbreak response and health systems management (120).

AI holds the potential to reduce the profound impact of IPV on women's lives by transforming the management and mitigation of its psychological and biological long-term health effects through early detection, prevention, support, and innovative research (121, 122).

AI algorithms, particularly those used in machine learning, can process large datasets of biomarkers from IPV survivors and control groups to identify patterns and specific biomarkers associated with stress and trauma (123, 124). These biomarkers could act as early indicators of IPV's biological impact, potentially leading to the development of new diagnostic tools (125, 126). AI can also combine biomarker data with other biological, psychological, and social information to create a comprehensive model of how IPV impacts health. This approach could help identify key factors influencing the long-term health consequences of IPV and guide targeted interventions.

Additionally, AI can support the development of personalized treatment plans by analyzing specific EV signatures of individuals. This allows unique biological changes resulting from IPV to be specifically addressed. AI-driven analysis of EV profiles over time may help monitor a survivor's response to treatment, allowing adjustments to improve therapeutic outcomes (127, 128).

In terms of predictive modeling and risk assessment, AI can identify EV-based biomarkers that predict the likelihood of developing chronic conditions such as PTSD or cardiovascular disease following IPV, providing opportunities for early interventions for those at increased risk of serious long-term effects (129, 130). AI tools can also model how EV-mediated communication might influence epigenetic changes over time, offering insights into how IPV-related stress may lead to long-term changes in gene expression and health outcomes (131, 117).

By analyzing a range of data sources, such as healthcare records, social media activity, and wearable devices, AI algorithms can identify patterns that may indicate that a person is at risk of experiencing psychological stress (132–135). Similarly, by assessing changes in an individual's communication patterns or social interactions, AI can recognize signs of distress or isolation that are often associated with depression (136). AI-integrated wearable devices can track stress levels in real-time, providing feedback and interventions, such as relaxation exercises, when needed (137, 138). Predictive analytics, leveraging various risk factors such as a history of abuse, substance use, or economic stressors, can help predict the likelihood of future violence, thus enabling more targeted and proactive interventions.

## 9 Discussion

IPV is a common and potentially devastating problem affecting women across the lifespan. Despite the increasing focus on primary and basic prevention of IPV, achieving this goal remains extremely challenging. Available data likely underestimate the true scale of the issue, as women who have experienced violence from their partners are often hesitant to report these incidents, even to health professionals.

Individual-level risk factors for IPV include nonwhite identities, lower educational attainment (less than high school), unwanted or unplanned pregnancies, substance use, history of child abuse, adolescent antisocial behaviors, and traditional gender role attitudes (139). The Centers for Disease Control and Prevention (CDC) further identifies young age, history of depression or suicide attempts, and economic insecurity as additional individual risk factors (140). Relationship factors include single-parent households, cohabiting relationships, relationship conflict (including jealousy or possessiveness), maladaptive dominance and control patterns, and low socioeconomic status (141).

The findings from this narrative review provide a deep understanding of the pervasive and enduring impact of IPV on survivors, highlighting both the psychological and biological consequences of this form of abuse.

The psychological consequences of IPV are particularly severe, with survivors often experiencing a range of mental health disorders, including PTSD, depression, and anxiety. PTSD is especially prevalent among IPV survivors, with rates significantly exceeding those in the general population. This heightened prevalence can be attributed to the chronic and pervasive nature of the trauma experienced in IPV, where the perpetrator is often someone the victim knows intimately, leading to profound feelings of betrayal and fear.

PTSD in IPV survivors is characterized by symptoms such as flashbacks, nightmares, hypervigilance, and emotional numbness. These symptoms can be debilitating, interfering with daily functioning and significantly diminishing quality of life. Depression and anxiety are also common, with survivors often experiencing persistent feelings of sadness, hopelessness, and excessive worry. These mental health conditions not only impact emotional well-being but also affect physical health, social relationships, and the ability to maintain employment or pursue education.

Beyond the psychological effects, this review delves into the emerging field of epigenetics, exploring how the stress and trauma associated with IPV can lead to biological changes at the molecular level. Epigenetics refers to changes in gene expression that do not involve alterations to the DNA sequence itself but are influenced by environmental factors such as trauma and stress. One of the most studied mechanisms in this context is DNA methylation, where methyl groups are added to the DNA molecule, often leading to the suppression of gene activity. IPV can lead to epigenetic modifications in genes associated with the stress response, such as the NR3C1 and the MAOA genes. These modifications can result in an altered stress response, making survivors more susceptible to stress-related disorders like PTSD and depression. Moreover, these epigenetic changes can potentially be inherited by future generations, meaning that the impact of IPV could extend beyond the immediate victim, affecting descendants and perpetuating a cycle of trauma and vulnerability.

Epigenetic changes due to episodes of violence or psychological trauma, like other forms of epigenetic changes, can be reversible, but their reversibility depends on various factors (142). Firstly, the duration and intensity of the trauma play a crucial role: prolonged or very severe trauma may leave more stable epigenetic marks, whereas less severe trauma may induce more easily reversible epigenetic changes (143). Another important factor is the age of the person at the time of the trauma: epigenetic changes in children, especially during brain development, tend to have more lasting effects than those in adults. However, there is some evidence that reprogramming-induced rejuvenation strategies have begun to greatly alter longevity research not only to tackle age-related defects but also to possibly reverse the cellular ageing process (144).

IPV also has broader health implications, as survivors are at increased risk for chronic conditions such as cardiovascular disease, chronic pain, and immune system dysfunction. These health issues are believed to be, at least in part, a consequence of the prolonged stress and trauma experienced by survivors, leading to systemic inflammation and dysregulation of the body's stress-response systems.

**Figure 1:**
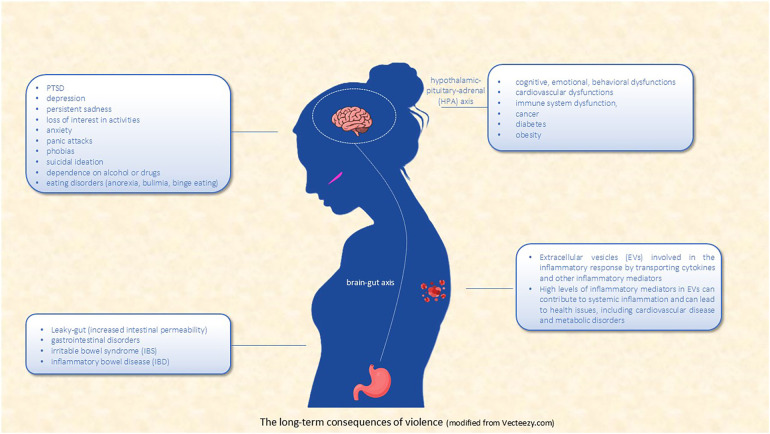
The long-term consequences of violence (modified from vecteezy.com).

Given the profound impact of IPV on both mental and physical health (Figure 1), there is a critical need for early intervention and comprehensive support systems. Immediate intervention is crucial in preventing the long-term consequences of IPV, as addressing trauma soon after it occurs can help mitigate its effects on both mental health and biological functioning. This can be achieved through psychological support, such as counseling and therapy, and medical care that addresses physical injuries or health concerns.

The above highlights the importance of an integrated healthcare approach that considers the complex interplay between the psychological, biological, and social aspects of IPV. Survivors often require a multidisciplinary team of healthcare providers, including mental health professionals, primary care physicians, and social workers, to address their diverse needs. Additionally, support systems that provide safe housing, legal assistance, and financial support are essential for helping survivors rebuild their lives and escape the cycle of violence.

Environmental factors such as a strong support network or healthy lifestyles may also contribute to restoring a healthier epigenetic state (145). Furthermore, research is underway on experimental drugs that aim to reverse epigenetic changes linked to trauma and mental disorders, potentially opening new therapeutic avenues for the future (146).

A further interesting aspect concerns the possibility that trauma-induced epigenetic modifications may be passed on to subsequent generations. Some studies suggest that these modifications may pass through the germ line, i.e., through eggs or spermatozoa, and thus influence offspring (147, 148). However, even in these cases, the transmitted epigenetic modifications could potentially be reversible if the subject is exposed to a favorable environment and positive treatment.

Continued research on the long-term effects of IPV and the development of targeted interventions is crucial. Longitudinal studies are particularly important, as they can provide valuable insights into how the effects of IPV evolve over time and how different interventions can alter these trajectories. Such research can inform policy decisions, leading to the implementation of more effective prevention and support programs.

Finally, promoting greater awareness and education about the impacts of IPV, both among health professionals and the public, allows for better and earlier recognition of the signs of violence and an understanding of its profound effects. This awareness can enable society to intervene earlier and support survivors more effectively.

Given the profound consequences of IPV, it is essential to implement early interventions and integrated care approaches. In addition to psychological and medical support, targeted social interventions including legal assistance, economic support, and access to safe housing resources are needed. Future research should focus on identifying epigenetic biomarkers that could enable early diagnosis and personalized interventions aimed at reducing the long-term effects of IPV.

## 10 Limitations

Although ChatGPT-4 significantly improved the efficiency of our literature review process, we acknowledge several limitations in its use. ChatGPT-4 does not have direct access to academic databases such as PubMed, meaning that all AI-assisted searches were conducted using manually retrieved articles. Additionally, AI models like ChatGPT-4 can sometimes generate inaccurate or misleading summaries, a phenomenon known as “hallucination.” To mitigate this risk, all AI-generated outputs were carefully verified against the original articles by the authors. Another limitation is the potential for bias, as ChatGPT-4 prioritizes text patterns from its training data, which may emphasize frequently discussed topics while underrepresenting less-cited but equally relevant research. Furthermore, although ChatGPT-4 efficiently summarizes and categorizes literature, it does not replace the human ability to critically assess the quality, methodology, and limitations of individual studies. To ensure scientific rigor, all final literature selections and evaluations were performed manually by the research team, independent of AI-generated suggestions.

Despite our efforts to provide a comprehensive review of the long-term health consequences of intimate partner violence (IPV), this study has further limitations other than above mentioned. First of all, as a narrative review, it does not adhere to the strict methodological framework of a systematic review, which typically involves predefined inclusion and exclusion criteria, risk-of-bias assessment, and statistical meta-analysis. While the narrative approach allowed for greater flexibility in integrating findings across diverse disciplines—including psychology, neuroscience, and molecular biology—it also introduces a higher risk of selection bias, as study inclusion was based on relevance rather than a predefined systematic process. A further limitation relates to potential biases in the individual studies included. Many of the studies reviewed come from different fields with varying methodological approaches, sample sizes, and definitions of IPV. Some studies rely on self-reported data, which may be subject to recall bias or underreporting due to the stigma associated with IPV. Additionally, while we aimed to include research from a wide range of populations, certain geographic or socio-cultural contexts may be underrepresented, limiting the generalizability of our findings. Future research should consider conducting systematic meta-analyses to quantify the strength of associations between IPV and stress-related disorders while accounting for potential confounders such as socioeconomic status, cultural background, and access to healthcare.

Another limitation is the lack of longitudinal data in many of the studies reviewed. While our paper discusses the long-term health effects of IPV, much of the available literature consists of cross-sectional studies that capture data at a single point in time. This limits our ability to establish causality between IPV exposure and biological or psychological outcomes. More longitudinal studies are needed to track IPV survivors over time, assess changes in biological markers such as epigenetic modifications and microbiome shifts, and evaluate the long-term efficacy of intervention strategies.

Finally, while we discuss emerging areas such as extracellular vesicles (EVs) as potential biomarkers of IPV resilience, this remains an underexplored field with limited clinical validation. Many findings in this domain are based on preclinical models or small-scale pilot studies, necessitating further large-scale human research to confirm their relevance. The integration of AI-driven analysis in epigenomics and biomarker research holds promise for identifying at-risk individuals, but its application in IPV-related health outcomes is still in its infancy. Future research should explore precision medicine approaches that combine molecular, psychological, and behavioral data to develop more personalized intervention strategies for IPV survivors.

Despite these limitations, we believe that our review provides a valuable synthesis of current knowledge, offering a multidisciplinary perspective on the biological and psychological consequences of IPV. By identifying key gaps in the literature, we hope this work will serve as a foundation for future research and policy initiatives aimed at improving prevention, early detection, and treatment of IPV-related health consequences.

## 11 Conclusion

The effects of IPV can be deep and long-lasting, impacting the victim's physical, emotional, and psychological well-being. While it is easier to assess the short-term consequences of IPV (physical injuries, behavioral changes, health issues and economic impact), much more complex is the assessment of its long-term impact.

In recent years, research found evidence that IPV exposure significantly affects by increasing the risk of adverse outcomes such as depression, suicidal thoughts and attempts, anxiety, and PTSD (12, 149). The severity and nature of these effects can vary depending on the duration and intensity of the abuse, the victim's resilience and support systems, and access to healthcare and support services.

Despite this, to our knowledge there are few studies on the long-term consequences of IPV. To fill this gap, the Italian Ministry of Health financed the multicentric and transdisciplinary project “The Violence against women: long-term health effects for precision prevention”, for supporting women and at creating new territorial models and innovative strategies to counteract long-term health effects (150).

This project aims to integrate the databases to establish a record-linkage to allow records from different archives to be compared and complemented. In fact, the interconnection of the different data flows could allow for more precise delineation of a woman's health or illness profile allowing for the implementation of preventive interventions for health-related consequences of violence. This project, therefore, is in the groove of a new generation of longitudinal studies characterized by the collection of properly preserved biological samples and improved questionnaires (including the collection of social variables) that using new technologies will characterize individual “environmental” exposures, such as including violence, to offer precision prevention interventions.

The ambition of the project is also to lay the foundation for the study of long-term consequences of IPV. Indeed, survivors of IPV are at higher risk for chronic conditions such as cardiovascular disease, chronic pain, gastrointestinal disorders, and other stress-related illnesses (151–154). We strongly believe that new scientific approaches based on clinical molecular research in parallel with social, educational, clinical and health care interventions represent a new and mandatory way to achieve innovative precision prevention protocols and resilience. The parallel tracking of different biomarkers could represent a powerful approach, enhanced with AI, for PTSD epigenomic research for limiting the negative long-term effects of IPV. Accordingly, a major aim of our project is to identify biomarkers of the disorder useful to distinguish subjects at high and low risk of developing PTSD for targeting specific prevention protocols.

To link violence to the early onset of some non-communicable diseases, it is necessary to build on the overall health history of women.

An important premise of our project is the creation of a unique personal code that will allow us to establish a personal clinical history for each patient.

Early detection of trauma-induced chronic and non-communicable diseases is crucial for a better healthy life.
