# Supplementary material for: Intimate partner violence and stress-related disorders: from epigenomics to resilience
Source: Front Glob Womens Health. 2025 May 12;6:1536169. doi: 10.3389/fgwh.2025.1536169 (PMC12104246; doi:10.3389/fgwh.2025.1536169)
Supplement: Supplementary file 1 [file Table1.docx]

**Intimate partner violence and stress-related disorders: from epigenomics to resilience**

**Supplementary Material**

Custom-made search terms list by key areas:

**Core Themes**

- Intimate Partner Violence (IPV)
- Gender-based violence
- Domestic violence and health
- Psychological trauma and IPV
- Long-term effects of IPV

**Psychological and Psychiatric Impacts**

- IPV and PTSD (Post-Traumatic Stress Disorder)
- IPV and depression
- Anxiety disorders and intimate partner violence
- Dissociation and trauma
- Suicidal ideation in IPV survivors

**Biological and Molecular Mechanisms**

- Epigenetics of trauma
- DNA methylation and IPV
- HPA axis dysregulation and stress
- Neurotransmitter imbalance and IPV
- Oxidative stress and PTSD

**Microbiome and Brain-Gut Axis**

- Microbiome and psychological trauma
- Gut-brain axis and stress resilience
- Intestinal permeability and stress disorders
- Chronic stress and microbiome diversity

**Extracellular Vesicles and Biomarkers**

- Extracellular vesicles and PTSD
- Biomarkers of stress-related disorders
- Blood-brain barrier dysfunction in trauma
- miRNA signatures in IPV survivors

**Sleep Disturbances and IPV**

- Insomnia and PTSD
- Circadian rhythm disruption and trauma
- Sleep disorders in IPV survivors
- Melatonin and stress resilience

**Artificial Intelligence and IPV Research**

- AI in mental health research
- Machine learning for PTSD detection
- AI-based literature screening
- Big data in IPV research

**Public Health and Intervention Strategies**

- Resilience in IPV survivors
- Precision medicine in trauma research
- Public health approaches to IPV
- Prevention of stress-related disorders

These search terms have been combined using Boolean operators (AND, OR, NOT) for more specific results.
